# Comparison of multi-parallel qPCR and double-slide Kato-Katz for detection of soil-transmitted helminth infection among children in rural Bangladesh

**DOI:** 10.1371/journal.pntd.0008087

**Published:** 2020-04-24

**Authors:** Jade Benjamin-Chung, Nils Pilotte, Ayse Ercumen, Jessica R. Grant, Jacqueline R. M. A. Maasch, Andrew M. Gonzalez, Ashanta C. Ester, Benjamin F. Arnold, Mahbubur Rahman, Rashidul Haque, Alan E. Hubbard, Stephen P. Luby, Steven A. Williams, John M. Colford

**Affiliations:** 1 Division of Epidemiology & Biostatistics, University of California, Berkeley, Berkeley, California, United States of America; 2 Department of Biological Sciences, Smith College, Northampton, Massachusetts, United States of America; 3 Molecular and Cellular Biology Program, University of Massachusetts, Amherst, Massachusetts, United States of America; 4 Department of Forestry and Environmental Resources, North Carolina State University, Raleigh, North Carolina, United States of America; 5 Infectious Disease Division, International Centre for Diarrhoeal Disease Research, Bangladesh (icddr,b), Dhaka, Bangladesh; 6 Infectious Diseases & Geographic Medicine, Stanford University, Stanford, California, United States of America; Emory University, UNITED STATES

## Abstract

There is growing interest in local elimination of soil-transmitted helminth (STH) infection in endemic settings. In such settings, highly sensitive diagnostics are needed to detect STH infection. We compared double-slide Kato-Katz, the most commonly used copromicroscopic detection method, to multi-parallel quantitative polymerase chain reaction (qPCR) in 2,799 stool samples from children aged 2–12 years in a setting in rural Bangladesh with predominantly low STH infection intensity. We estimated the sensitivity and specificity of each diagnostic using Bayesian latent class analysis. Compared to double-slide Kato-Katz, STH prevalence using qPCR was almost 3-fold higher for hookworm species and nearly 2-fold higher for *Trichuris trichiura*. *Ascaris lumbricoides* prevalence was lower using qPCR, and 26% of samples classified as *A*. *lumbricoides* positive by Kato-Katz were negative by qPCR. Amplicon sequencing of the 18S rDNA from 10 samples confirmed that *A*. *lumbricoides* was absent in samples classified as positive by Kato-Katz and negative by qPCR. The sensitivity of Kato-Katz was 49% for *A*. *lumbricoides*, 32% for hookworm, and 52% for *T*. *trichiura*; the sensitivity of qPCR was 79% for *A*. *lumbricoides*, 93% for hookworm, and 90% for *T*. *trichiura*. Specificity was ≥ 97% for both tests for all STH except for Kato-Katz for *A*. *lumbricoides* (specificity = 68%). There were moderate negative, monotonic correlations between qPCR cycle quantification values and eggs per gram quantified by Kato-Katz. While it is widely assumed that double-slide Kato-Katz has few false positives, our results indicate otherwise and highlight inherent limitations of the Kato-Katz technique. qPCR had higher sensitivity than Kato-Katz in this low intensity infection setting.

## Author summary

Soil-transmitted helminth infections (STH) (e.g., *Ascaris*, hookworm, *Trichuris*) contribute to a large burden of disease among children in low- and middle-income countries. There is increasing interest in implementing large-scale deworming programs to eliminate STH in certain settings. Efforts to monitor whether local elimination has occurred require sensitive diagnostic tests that will not miss positive cases. Kato-Katz, a microscopy-based diagnostic test, has commonly been used to identify STH eggs in stool, but in settings where infection intensity is low, this method frequently misses positive samples because it requires visual identification of small numbers of eggs, and hookworm eggs may degrade prior to visualization. Quantitative polymerase chain reaction (qPCR) is a molecular diagnostic method for detecting STH. It may detect more low intensity infections than Kato-Katz because it identifies STH DNA in stool; DNA can be detected in very small quantities and is less likely to degrade than STH ova. Thus, qPCR is likely to be more accurate than Kato-Katz. This study compared the performance of double-slide Kato-Katz and qPCR using 2,799 stool samples from children aged 2–12 years in a setting in rural Bangladesh with predominantly low STH infection intensity. qPCR was more sensitive than Kato-Katz for hookworm and *Trichuris* infections. 26% of samples were classified as *Ascaris* positive by Kato-Katz and negative by qPCR. DNA sequencing of 10 samples confirmed that *Ascaris* was absent in samples classified as positive by Kato-Katz and negative by qPCR. We conclude that Kato-Katz likely produced false positive results for *Ascaris* and that qPCR had a higher sensitivity than double-slide Kato-Katz in this low infection intensity setting.

## Introduction

Soil-transmitted helminths (STH) infect an estimated 1.5 billion individuals, almost one fifth of the global population [1]. These infections contribute to a substantial burden of disability and disease, particularly for children [2]. To control the burden of STH infection, mass drug administration programs deliver preventive chemotherapy to populations at risk, such as school children. In recent years, approximately 33% of children in endemic settings received deworming medication for STH in the past six months according to caregiver report [3]. Such programs have expanded dramatically following the London Declaration on Neglected Tropical Diseases in 2012. Mass drug administration (MDA) programs have yielded large reductions in STH infection prevalence and intensity to date [4]. While the World Health Organization has set goals for STH focused on morbidity control [5], there is an active body of research focused on whether local elimination of STH is possible in endemic settings through community-wide MDA for deworming [6–9]. As STH infection intensity and prevalence wane, increasingly sensitive diagnostic methods are required to determine whether STH transmission has been interrupted and when interventions can be scaled back or discontinued [10].

Diagnostic methods for STH include copromicroscopic methods, such as the Kato-Katz method [11,12], formol-ether concentration [13], FLOTAC [14], mini-FLOTAC [15–18], and FECPAK^G2^ [18,19]. Of these, Kato-Katz is the most commonly used diagnostic method for STH surveillance because it is inexpensive and relatively easy to perform in low-resource settings [20]. A significant limitation of this method is that samples must be evaluated within an hour of preparation in order to detect fragile hookworm ova. In addition, *Necator americanus* and *Ancylostoma duodenale* cannot be distinguished using Kato-Katz because of their morphological similarities. Because egg excretion is highly variable over time [21], there can be substantial variation in the count of eggs per gram (epg) of stool collected on different days. Kato-Katz has improved sensitivity when performed on multiple samples from defecation events on different days, but such samples are often logistically prohibitive to collect. Double-slide Kato-Katz examines stool using two separate aliquots from a single defecation event prepared as separate slides in order to help reduce the variability and increase sensitivity. Furthermore, a meta-analysis estimated the sensitivity of double-slide Kato-Katz to be 64.6% for *Ascaris lumbricoides*, 84.8% for *Trichuris trichiura*, and 63.0% for hookworm [22]. Another limitation of Kato-Katz is that its sensitivity is lower in low infection intensity settings (55.2% for *A*. *lumbricoides*, 79.8% for *T*. *trichiura*, and 52.6% for hookworm) [22]. Alternative copromicroscopic STH diagnostic methods, such as formol-ether concentration, FLOTAC, mini-FLOTAC, and the McMaster method also have moderate to low sensitivity for different types of STH in low-intensity settings [22].

Multiplex [23,24] and multi-parallel [25,26] quantitative polymerase chain reaction (qPCR) assays have been developed to detect STH DNA in stool. Multiplex and multi-parallel assays allow for detection of multiple helminths from a single stool sample. Growing evidence suggests that these methods may be used to estimate infection intensity, although qPCR-based cutoffs for light, moderate, and heavy intensity infections have not yet been defined [24,27,28]. To our knowledge, six studies have compared diagnostic performance of copromicroscopic methods and qPCR; these studies reported that analyses classifying STH status using qPCR as a diagnostic tool showed higher prevalence compared to those using single and/or double-slide Kato-Katz because of the higher sensitivity of the qPCR [18,23,25,27–29].

The greater sensitivity, specificity, and precision of qPCR relative to Kato-Katz makes it an attractive diagnostic for monitoring the successes of STH control programs deploying mass deworming since infection intensity tends to decline after several years of intervention [30]. As a population reaches an STH transmission breakpoint, worm burdens decrease, and the comparatively low sensitivity of Kato-Katz may preclude its use in monitoring STH prevalence. In addition, a growing body of work suggests that qPCR may also be used to estimate infection intensity; one study found that qPCR is approximately 3.6 times more precise than Kato-Katz in estimating *A*. *lumbricoides* egg intensity [31]. Such additional precision could increase the statistical power available to detect whether the STH prevalence meets the threshold for elimination.

The objectives of this study were to estimate the prevalence of STH infection and infection intensity using double-slide Kato-Katz and multi-parallel qPCR and to estimate the sensitivity and specificity of each method using Bayesian latent class models and archived samples collected from children aged 2–12 years in rural Bangladesh.

## Methods

### Ethics statement

The study protocol was approved by the Ethical Review Committee at icddr,b (PR-14105), the Committee for the Protection of Human Subjects at the University of California, Berkeley (2014-08-6658), and the institutional review board at Stanford University (27864). Prior to enrollment, all adult subjects provided written informed consent. Parents or guardians of children provided written informed consent on behalf of children.

### Study population

This study analyzed archived stool samples that were collected from participants in the WASH Benefits Bangladesh trial between May 2015 and May 2016 (Clinicaltrials.gov NCT01590095) [32,33]. The trial was implemented in Gazipur, Mymensingh, Tangail and Kishoreganj districts of Bangladesh. Biannual school-based mass drug administration with mebendazole had been implemented nationally in Bangladesh since 2008 and was ongoing in the study area during the trial. The trial delivered interventions to improve water, sanitation, handwashing, and nutrition and included single and combined intervention arms. Full details of interventions and user adherence have been published elsewhere [33,34]. This study randomly selected 2,800 stool specimens collected from children aged 22 months to 12 years of age (mean = 57 months) enrolled in the individual improved water, sanitation, handwashing, and combined water+sanitation+handwashing (WSH) arms. This sample size was chosen in order to estimate sensitivity with precision of +/- 5% and 80% statistical power; we assumed STH prevalence estimates from a prior study in rural Bangladesh [35] and double-slide Kato-Katz sensitivity and specificity estimates from a prior meta-analysis of STH diagnostic accuracy [22].

### Stool collection and storage

Field staff provided study participants’ primary caregivers with sterile containers and requested that they collect stool from the child’s defecation event the next morning. Field staff returned to the house at least three times to attempt to collect stool containers. As part of the WASH Benefits trial, all study participants received a single dose of albendazole following stool collection. Field staff transported stool specimens on ice to the field laboratory, where 1 g of stool was archived in 1 ml of 100% ethanol. Specimens were stored at -20°C prior to relocation to a -80°C freezer. In the WASH Benefits parasites sub-study, the median time that elapsed between the reported time of defecation and when the stool was first placed on ice was 2.4 hours (range: 0.03 to 18 hours), and the median time that elapsed between the date of specimen collection and the date when specimens were stored in a -80° C freezer was 11 days (range: 1 to 330 days).

### Kato-Katz procedures

Technicians were trained in double-slide Kato-Katz using the Vestergaard Frandsen protocol at the International Centre for Diarrhoeal Disease Research, Bangladesh (icddr,b) parasitology laboratory. On the day of stool collection, trained technicians performed double-slide Kato-Katz [11,12] on fresh stool within 30 minutes of preparing each sample. For quality assurance, 10% of slides were evaluated independently by two technicians, and 5% were evaluated by the same experienced parasitologist who conducted Kato-Katz training. The quality assurance results have previously been published elsewhere [34]. In brief, agreement between laboratory technicians was high (Kappa statistic > 0.99 for each STH). The Kappa statistic for agreement between laboratory technicians and experienced parasitologists was 0.92 for *A*. *lumbricoides*, 0.20 for hookworm, and 0.86 for *T*. *trichiura*. The agreement is likely lower for hookworm because experienced parasitologists examined slides up to a few days after laboratory technicians, and hookworm ova may have begun to disintegrate by that time. For the subset of samples for which quality control was performed on the same day, the agreement for hookworm was high. Agreement decreased as the time between the original assessment and the senior parasitologist’s assessment increased (S1 Table). Samples in which at least one STH egg was visualized during Kato-Katz were classified as positive. Kato-Katz technicians did not have access to any clinical information about study participants.

### DNA isolation and qPCR procedures

Approximately 1–2 years after stool collection, preserved stool specimens were shipped to Smith College in Northampton, MA, United States for qPCR analyses. Prior to shipment, ethanol was evaporated from all samples for compliance with shipping regulations, and samples were shipped on dry ice. Upon receipt at Smith College, all samples were stored at -20°C until DNA extraction. DNA was extracted using the FastDNA Spin Kit for Soil (MP Biomedicals, Solon, OH) utilizing a previously published modified version of the manufacturer’s methodology [25]. Following DNA extraction and prior to qPCR analyses, extractions were stored at -20°C. We assigned each sample a unique number following extraction, and this number was electronically linked to the initial barcode on the stool sample tube at the time of extraction. Results were then linked back to the initial sample using both the Smith-assigned number and the barcode, and samples underwent qPCR processing in the same batches that they were extracted in. An internal amplification control (IAC) plasmid was employed during the extraction of each sample to ensure successful isolation, and adequate recovery of DNA [36]. Utilizing previously described reaction methods [37], any sample which failed to produce a positive qPCR result for one or more of the IAC replicates underwent re-extraction. Similarly, following the completion of all extractions, the mean quantitation cycle (Cq) value for the IAC results from all samples was calculated, as was the standard deviation of the mean. Any sample with an individual IAC result determined to be 3 or more standard deviations greater than the mean underwent re-extraction, as such a recovery was deemed suboptimal. Following re-extraction, only the extracted DNA sample producing a positive IAC result within the defined Cq range (not the previously conducted invalid extract) was used for experimental testing.

Experimental qPCR reactions were performed using previously published multi-parallel assays targeting non-coding repetitive sequences to detect *Ascaris lumbricoides* [38], *Trichuris trichiura*, *Strongyloides stercoralis*, *Necator americanus*, *Ancylostoma duodenale* [26] and *Ancylostoma ceylanicum* [39] (S2 Table). Laboratory staff were blinded to Kato-Katz results for each sample during the initial qPCR analyses. However, upon completion of initial testing, all samples determined to be Kato-Katz positive but qPCR negative for *A*. *lumbricoides* were tested using a second qPCR assay targeting an unrelated, ribosomal DNA sequence in order to validate the negative qPCR result and confirm the absence of *A*. *lumbricoides* DNA [26,40].

For each assay, all samples were tested in replicate reactions and samples were classified as positive when amplification occurred in both reactions with a Cq value of <40, consistent with prior studies [41]. Samples which produced Cq values ≥40 in both replicate reactions or failed to amplify in both replicate reactions were classified as negative. Consistent with common practice [42], samples that were positive in one of two replicate reactions were re-tested in duplicate and classified as positive if the Cq value was <40 in at least one of two re-test reactions. In all cases of re-testing, the Cq value reported as the sample’s final result was the re-test Cq value.

In addition to the IAC control, all experimental qPCR reaction plates were accompanied by the testing of a titration of the appropriate assay’s control plasmid. Plasmid controls for each target sequence were prepared as previously described [38]. Each plasmid contained a single copy of the corresponding assay’s target sequence and 20 pg, 200 fg and 2 fg of plasmid were tested in duplicate reactions. Following the completion of all testing, the mean Cq value, across all experimental plates, for each plasmid concentration was determined. Any plate which produced a Cq value for any concentration of plasmid that was 3 or more standard deviations greater than the mean calculated for all plates was retested in its entirety, and all results from that plate were considered to be invalid. Only results from valid plates were reported (S3 Table).

### Sequencing

To verify that Kato-Katz positive/qPCR negative samples were truly qPCR negative for *A*. *lumbricoides*, we performed sequencing on a subset of samples. Because <1% of samples tested negative for hookworm or *T*. *trichiura* using qPCR but positive using Kato-Katz, we only performed sequencing for samples with discordant *A*. *lumbricoides* classification. A subset of 10 samples was selected to undergo amplicon sequencing-based analysis. To facilitate the selection of samples, all samples that were double-slide Kato-Katz positive for the presence of *A*. *lumbricoides* but qPCR negative for *A*. *lumbricoides* were identified. From this list, a convenience sample (n = 7) was then selected, such that two of these samples would contain epg counts characteristic of moderate-intensity infections as determined by World Health Organization guidelines, while five samples would contain egg counts that were characteristic of light intensity infections [43]. All seven of the Kato-Katz positive samples were negative by qPCR using both *A*. *lumbricoides* assays. Additionally, three Kato-Katz negative samples were chosen for inclusion in this panel. Two of these samples were selected due to their status as *A*. *lumbricoides-*positive as determined by qPCR analysis, while a single sample was selected that was both Kato-Katz negative and qPCR negative to serve as an uninfected control.

Samples were prepared for sequencing using a modified version of the Earth Microbiome Project’s 18S Illumina Amplicon Protocol available at: http://www.earthmicrobiome.org/protocols-and-standards/18s/. This protocol utilizes primers targeting the variable region 9 (V9) of the eukaryotic small subunit (SSU) rDNA [44]. This protocol has been used in the analysis of helminth biodiversity in the gut of rats [45] and validated as a method for assessing global parasite diversity [46]. Briefly, the targets within each sample were amplified in triplicate 25 μl reactions using a uniquely barcoded reverse primer coupled with a conserved forward primer (S4 Table). A “mammalian blocking” primer, based on a previously described strategy, was also included in all reactions to minimize the prevalence of mammal-derived amplicons [47]. All reactions contained 10 μl of Platinum Hot Start PCR Master Mix (2X) (ThermoFisher, Waltham, MA), 0.2 μM forward and reverse primers, and 1.6 μM “mammalian blocking” primer (Integrated DNA Technologies, Coralville, IA). Cycling consisted of an initial denaturation step of 94°C for 3 minutes, followed by 35 cycles of 94°C for 45 seconds, 65°C for 15 seconds, 57°C for 30 seconds, and 72°C for 90 seconds. Samples then underwent a final extension step at 72°C for 10 minutes. Following amplification, triplicate reactions were combined, and pooled products were run on a 1.5% agarose gel to confirm the presence of a band of the expected size (~260 bp). 240 ng of each pooled product was then combined in preparation for sequencing, and this library was purified using the ZR-96 Clean & Concentrator purification kit (Zymo Research, Irvine, CA). The purified library was diluted to a 9 pM concentration, and 30% PhiX was added to improve diversity. Sequencing then occurred on the Illumina MiSeq platform, using a MiSeq Reagent Kit v2 (300-cycles) (Illumina, San Diego, CA).

Sequencing results were analyzed using the Quantitative Insights into Microbial Ecology 2 (QIIME2) pipeline [48]. Briefly, all interlaced read pairs which did not contain a valid barcode were filtered out and all read pairs whose best match was to bacterial 16S ribosomal sequence were also removed. This resulted in a list of reads exclusively matching eukaryotic 18S sequence. Reads with 99% or greater identity were then classified into operational taxonomic units (OTUs) and consensus sequences were built. Taxonomy was then assigned to all OTUs that had ≥ 95% identity to sequences within the Silva release 132 QIIME-compatible database.

### Statistical methods

All statistical analyses were pooled across intervention arms to ensure sufficient statistical power. Unless otherwise specified, analyses were conducted in R version 3.4.3. Data and replication scripts are available through the Open Science Framework (https://osf.io/agk6w/). The STARD Standard and Bayes Latent Class Models Checklists for this study are included in S1 Checklist and S2 Checklist.

### Assessment of quality of Kato-Katz slide readings

For the 2,800 samples included in this analysis, we estimated concordance between individual Kato-Katz technicians and expert technicians by comparing the classification of positive/negative status for each STH on each slide as well as the mean difference in eggs counted per slide.

### Prevalence and correlation between eggs per gram and Cq values

We estimated the prevalence of STH infection using each diagnostic method with robust sandwich standard errors to account for clustering at the village level [49]. We calculated the agreement between prevalence estimates from double-slide Kato-Katz and qPCR using a kappa statistic. We tested whether prevalence differed between the two diagnostic methods using the Wilcoxon matched-pairs signed rank test that conditioned on randomized block in the original trial [32,50]. To classify infection intensity, we used the World Health Organization cutoffs (light intensity infections were defined as < 5,000 epg for *A*. *lumbricoides*, < 1,000 epg for *T*. *trichiura*, and < 2,000 epg for hookworm; moderate intensity infections were defined as 5,000 ≤ epg < 50,000 for *A*. *lumbricoides*, 1,000 ≤ epg < 10,000 for *T*. *trichiura*, and 2,000 ≤ epg < 4,000 for hookworm; heavy intensity infections were defined as ≥ 50,000 epg for *A*. *lumbricoides*, ≥ 10,000 epg for *T*. *trichiura*, and ≥ 4,000 epg for hookworm) [43]. To assess the correlation between epg estimated by double-slide Kato-Katz and Cq value estimated by qPCR, we estimated Kendall’s tau and calculated two-sided p-values using a bootstrap with 1,000 replicates that resampled blocks to account for the study’s cluster-randomized design using Stata version 14.2. We compared mean Cq values within levels of infection intensity using a paired t-test paired within randomized blocks.

### Estimation of sensitivity and specificity

A challenge in studies assessing accuracy of STH diagnostics is the lack of a gold standard diagnostic method [51]. We thus used two different approaches to estimate sensitivity and specificity of each method, consistent with prior studies. We created a reference using both detection methods and defined positivity as qPCR Cq values < 40 or at least one egg detected by double-slide Kato-Katz. We also used Bayesian latent class analysis, which defines the true prevalence of STH infection, sensitivity, and specificity as latent variables that are estimated simultaneously from the data and assumes no gold standard [22,28,52–54]. We hypothesized that the number of STH worms in the intestines influences STH DNA concentration and epg in stool, each of which determines observed STH infection measured by qPCR and Kato-Katz. Because observed STH status measured by qPCR and Kato-Katz each depends upon true worm burden, we defined a model that allowed for conditional dependence between each test by including terms for covariance between diagnostic methods since both methods are dependent upon the same underlying biological process [52] (S1 Text).

Consistent with prior studies, our model did not include separate latent variables for worm burden, DNA concentration and epg, which were unobserved, because the model would have been non-identifiable. Latent class models for assessing diagnostic accuracy of two diagnostic tests in the absence of a gold standard are not identifiable because the number of unknown parameters exceeds the degrees of freedom [55]; in this case, multiple unique parameter estimates can be produced from the same model, and a single set of estimates cannot be identified. Within a Bayesian framework, when informative prior distributions are defined for at least two parameters, the model is identifiable [56]. Consistent with prior studies, we used a highly informative prior for specificity of each diagnostic test to allow for identifiability [22,28,57,58] since prior studies have reported high specificity values for double-slide Kato-Katz [59,60] and qPCR [25,61] (S5 Table). For *A*. *lumbricoides*, we chose to specify a non-informative prior for double-slide Kato-Katz sensitivity and specificity and a less informative prior for qPCR sensitivity because of discordant test results (summarized below). Since the model’s parameter estimates are sensitive to the assumed prior distributions [62], we conducted a sensitivity analysis using alternative, more informative priors for *A*. *lumbricoides* (S6 Table). To estimate parameters, we used Monte Carlo simulation with 4 chains and 100,000 iterations, recording every 10^th^ result. We computed the mean and 2.5 and 97.5 percentiles of the posterior distribution using Gibbs sampling in WinBUGS version 14 [63].

## Results

We analyzed stool samples from 2,799 children using both double-slide Kato-Katz and qPCR. 51% of children were female, and 66% of children’s caregivers reported that they had received deworming in the past 6 months (S1 Fig). Concordance between individual Kato-Katz technicians and expert parasitologists in the classification of STH positive/negative status was close to 100% for all technicians and STH (S2 Fig). For *A*. *lumbricoides* and *T*. *trichiura*, kappa statistics comparing individual technicians and expert parasitologists were high, and the kappa statistic for hookworm was high when the expert parasitologist performed Kato-Katz on the same day as the original count (S7 Table). The difference in mean eggs per slide between individual Kato-Katz technicians and experts was < 60 for *A*. *lumbricoides*, < 15 for hookworm, and < 20 for *T*. *trichiura* (S3 Fig). We excluded the qPCR results of one sample from statistical analyses for all species because the internal amplification control was > 3 standard deviations from the mean upon initial testing and following re-extraction.

**Fig 1:**
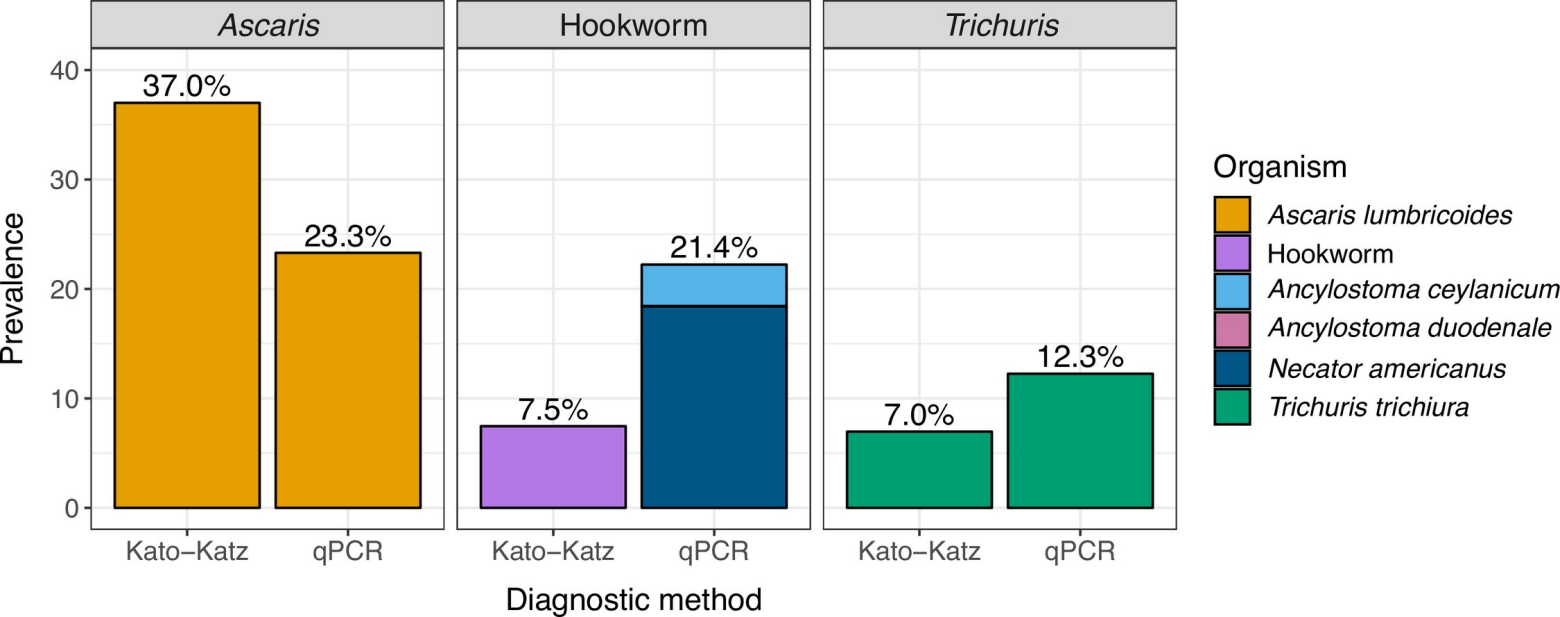
Observed soil-transmitted helminth prevalence by Double-Slide Kato-Katz and qPCR. Prevalence was estimated from stool samples collected from children aged 2–12 years in rural Bangladesh (N = 2,799).

**Fig 2:**
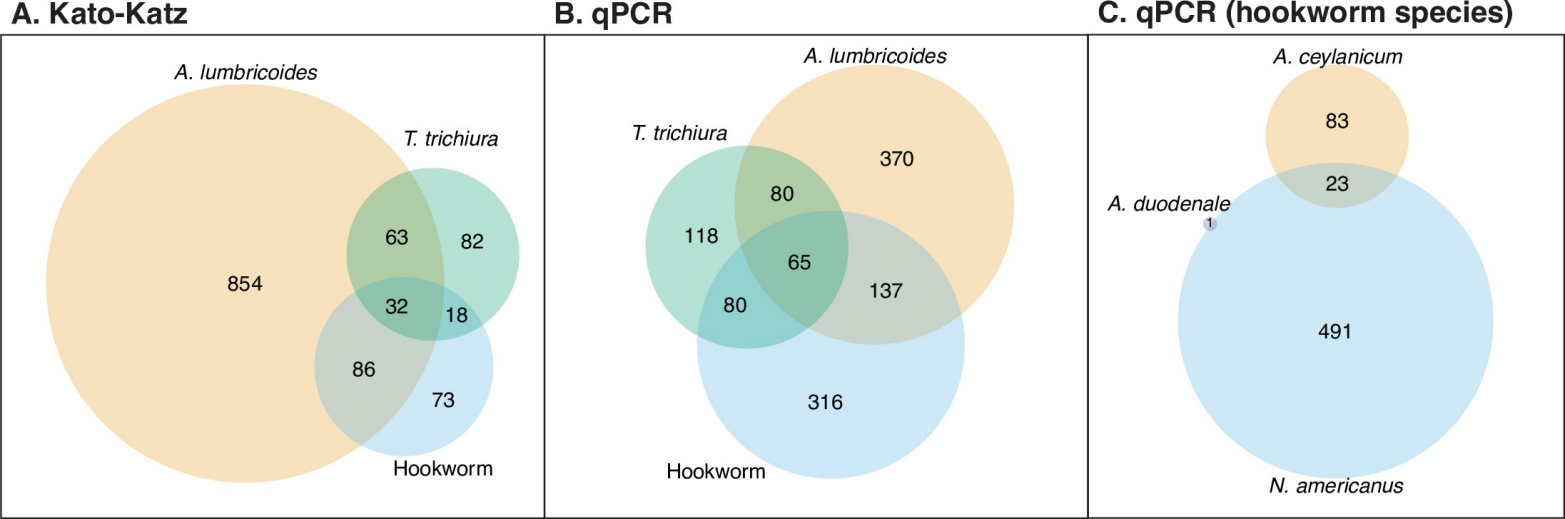
Venn diagram of co-infections detected by Double-Slide Kato-Katz and qPCR. Soil-transmitted helminth ova or DNA were detected in stool samples collected from children aged 2–12 years in rural Bangladesh using Kato-Katz or multi-parallel qPCR (N = 2,799).

The observed prevalence was higher for qPCR than double-slide Kato-Katz for hookworm and *T*. *trichiura* but lower for *A*. *lumbricoides* (Table 1, Fig 1). The observed prevalence of *A*. *lumbricoides* was 37.0% (95% CI 34.7%, 39.3%) using Kato-Katz and 23.3% (95% CI 20.9%, 25.7%) using qPCR. The observed prevalence of hookworm was 7.5% (95% CI 6.3%, 8.7%) using Kato-Katz. Using qPCR the observed prevalence of *Necator americanus* was 18.4% (95% CI 16.5%, 20.3%), and the observed prevalence of *Ancylostoma ceylanicum* was 3.8% (95% CI 2.9%, 4.6%); only one out of 2,799 samples tested positive for *Ancylostoma duodenale*. The observed prevalence of any hookworm species using qPCR was 21.4% (95% CI 19.4%, 23.3%). For *T*. *trichiura*, the observed prevalence was 7.0% (95% 5.6%, 8.3%) by Kato-Katz and 12.3% (95% CI 10.3%, 14.2%) by qPCR. Using Kato-Katz, 7% of children were infected with more than one STH, and using qPCR it was 13% (Fig 2). The observed prevalence of moderate intensity infection using Kato-Katz was 11% for *A*. *lumbricoides*, less than 1% for hookworm, and 5% for *T*. *trichiura*. The observed prevalence of heavy intensity infection using Kato-Katz was <1% for *A*. *lumbricoides*, 1% for hookworm, and 0% for *T*. *trichiura*. Using Kato-Katz, the geometric mean of epg was 5.14 for *A*. *lumbricoides* (134 among positives), 0.43 for hookworm (125 among positives), and 0.40 for *T*. *trichiura* (124 among positives).

**Table 1:** Observed soil-transmitted helminth prevalence, Double-Slide Kato-Katz eggs per gram, and qPCR Cq values (N = 2,799).

	Double-Slide Kato-Katz			qPCR		
	Number of positive samples	Observed prevalence (95% CI)	Median EPG in positive stool samples (range)	Number of positive samples	Observed prevalence (95% CI)	Median Cq value in positive stool samples (range)
*Ascaris lumbricoides*	1,035	37.0 (34.7, 39.3)	72 (12, 100,824)	652	23.3 (20.9, 25.7)	17.4 (4.4, 39.6)
Hookworm	209	7.5 (6.3, 8.7)	96 (24, 6,732)	598	21.4 (19.4, 23.3)	--
*Necator americanus*	--	--	--	515	18.4 (16.5, 20.3)	20.8 (13.9, 35.5)
*Ancylostoma ceylanicum*	--	--	--	106	3.8 (2.9, 4.6)	23.6 (14.4, 37.8)
*Ancylostoma duodenale*	--	--	--	1	< 0.001 (0.0, 0.10)	27.2 (27.2, 27.2)
*Trichuris trichiura*	195	7.0 (5.6, 8.3)	96 (24, 5,040)	343	12.3 (10.3, 14.2)	27.7 (21.4, 40.0)
*Strongyloides stercoralis*	--	--	--	17	0.6 (0.3, 0.9)	24.6 (19.1, 31.8)

Concordance between double-slide Kato-Katz and qPCR was higher for hookworm and *T*. *trichiura* than for *A*. *lumbricoides*. The p-value for permutation tests assessing whether the observed prevalence estimated by the two detection methods differed was < 0.001 for each STH (Table 2). 6–14% of samples classified as negative for the different species by Kato-Katz were positive using qPCR. 26% of samples classified as positive for *A*. *lumbricoides* by Kato-Katz were negative using qPCR. All samples determined to be Kato-Katz positive for *A*. *lumbricoides* but negative by qPCR analysis targeting the non-coding repeat also failed to produce a positive result when tested with the ITS-targeting assay. The majority of epg values among *A*. *lumbricoides* Kato-Katz-positive, qPCR-negative samples were < 100, although a small number of samples had epg between 1,000 and 50,000 (S4 Fig). The probability that an individual Kato-Katz technician classified a stool sample as *A*. *lumbricoides* positive among those classified as negative by qPCR ranged from 20% to 31%, and four out of six technicians were significantly more likely to misclassify a sample than the technician with the lowest misclassification rate (S5 Fig). There was no evidence that the proportion of samples classified as positive for *A*. *lumbricoides* by Kato-Katz and negative by qPCR (false positives assuming qPCR was the gold standard) followed a trend by date of Kato-Katz or date of DNA extraction (S6 Fig, S7 Fig); patterns by date were similar for false negatives (S8 Fig, S9 Fig).

**Table 2:** Classification of qPCR and Double-Slide Kato-Katz for each type of STH (N = 2,799).

*A*. *lumbricoides*	qPCR +	qPCR -	Kappa statistic (p-value)
Kato-Katz +	319 (11%)	716 (26%)	0.13 (< 0.001)
Kato-Katz -	333 (12%)	1431 (51%)	
**Hookworm**	**qPCR +**	**qPCR -**	
Kato-Katz +	195 (7%)	14 (1%)	0.42 (< 0.001)
Kato-Katz -	403 (14%)	2187 (78%)	
***T*. *trichiura***	**qPCR +**	**qPCR -**	
Kato-Katz +	167 (6%)	28 (1%)	0.58 (< 0.001)
Kato-Katz -	176 (6%)	2428 (87%)	

Using Bayesian latent class models, the estimated true prevalence was 27% (95% Bayesian Credible Interval (BCI) 20%, 37%) for *A*. *lumbricoides*, 20% (95% BCI 17%, 24%) for hookworm, and 11% (95% BCI 8%, 14%) for *T*. *trichiura*. For double-slide Kato-Katz, the sensitivity was 49% (95% BCI 34%, 64%) for *A*. *lumbricoides*, 32% (95% BCI 22%, 41%) for hookworm, and 52% (95% BCI 33%, 71%) for *T*. *trichiura*, and the specificity was 68% (95% BCI 61%, 77%) for *A*. *lumbricoides*, 99% (95% BCI 96%, 100%) for hookworm, and 98% (95% BCI 96%, 100%) for *T*. *trichiura* (Table 3). For qPCR, the sensitivity was 79% (95% BCI 61%, 99%) for *A*. *lumbricoides*, 93% (95% BCI 82%, 100%) for hookworm, and 90% (95% BCI 81%, 100%) for *T*. *trichiura*, and the specificity was 97% (95% BCI 95%, 100%) for all three STH. The sensitivity analysis for *A*. *lumbricoides* using alternative more informative priors produced similar estimates for Kato-Katz and higher sensitivity for qPCR; when more informative priors were used for both Kato-Katz and qPCR, the estimated sensitivity of qPCR was 90% (95% BCI 80%, 99%) (S8 Table). Pooling both methods as the gold standard, the sensitivity and specificity of Kato-Katz was similar to those estimated from the Bayesian model, and for qPCR the sensitivity of hookworm and *T*. *trichiura* was higher but the specificity was lower than estimated by the Bayesian model.

**Table 3:** Estimated sensitivity, and specificity of each diagnostic method using Bayesian latent class models.

	Double-Slide Kato-Katz		qPCR	
	Sensitivity (%) (95% BCI)	Specificity (%) (95% BCI)	Sensitivity (%) (95% BCI)	Specificity (%) (95% BCI)
**Bayesian latent class model**				
*Ascaris lumbricoides*	49 (34, 64)	68 (61, 77)	79 (61, 99)	97 (95, 100)
Hookworm^a^	32 (22, 41)	99 (96, 100)	93 (82, 100)	97 (95, 100)
*Trichuris trichiura*	52 (33, 71)	98 (96, 100)	90 (81, 100)	97 (95, 100)
**Any positive test (sensitivity) or any negative test (specificity) using Double-Slide Kato-Katz or qPCR**				
	Sensitivity (%) (95% CI)	Specificity (%) (95% CI)	Sensitivity (%) (95% CI)	Specificity (%) (95% CI)
*Ascaris lumbricoides*^b^	49 (45, 53)	67 (64, 69)	--	--
Hookworm^a^	34 (30, 38)	99 (99, 100)	98 (97, 99)	85 (83, 86)
*Trichuris trichiura*	53 (47, 58)	99 (99, 99)	92 (90, 95)	93 (92, 94)

95% BCI: 95% Bayesian credible interval

95% CI: 95% confidence interval

^a^ *Necator americanus*, *Ancylostoma duodenale*, and *Ancylostoma ceylanicum* combined

^b^ Due to the high discordance between qPCR and double-slide Kato-Katz for *A*. *lumbricoides*, we estimated sensitivity and specificity using qPCR alone as the gold standard.

**Fig 3:**
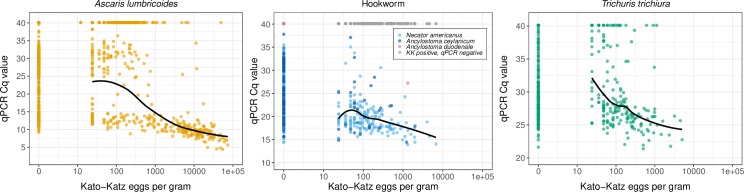
Relationship between Cq values measured by qPCR and eggs per gram estimated using Double-Slide Kato-Katz. Soil-transmitted helminth ova or DNA were detected by Kato-Katz and qPCR in stool samples collected from children aged 2–12 years in rural Bangladesh (N = 2,799). The black solid line is the LOESS smoother for values that were classified as positive using both tests. Gray points indicate results that were negative by qPCR but positive by Kato-Katz; they are gray because Kato-Katz cannot differentiate between hookworm species. The Kendall’s tau comparing eggs per gram and Cq values was -0.442 for *A*. *lumbricoides*, -0.346 for *N*. *americanus*, -0.266 for *A*. *ceylanicum*, and -0.248 for *T*. *trichiura* (for each, the p-value was < 0.001).

**Fig 4:**
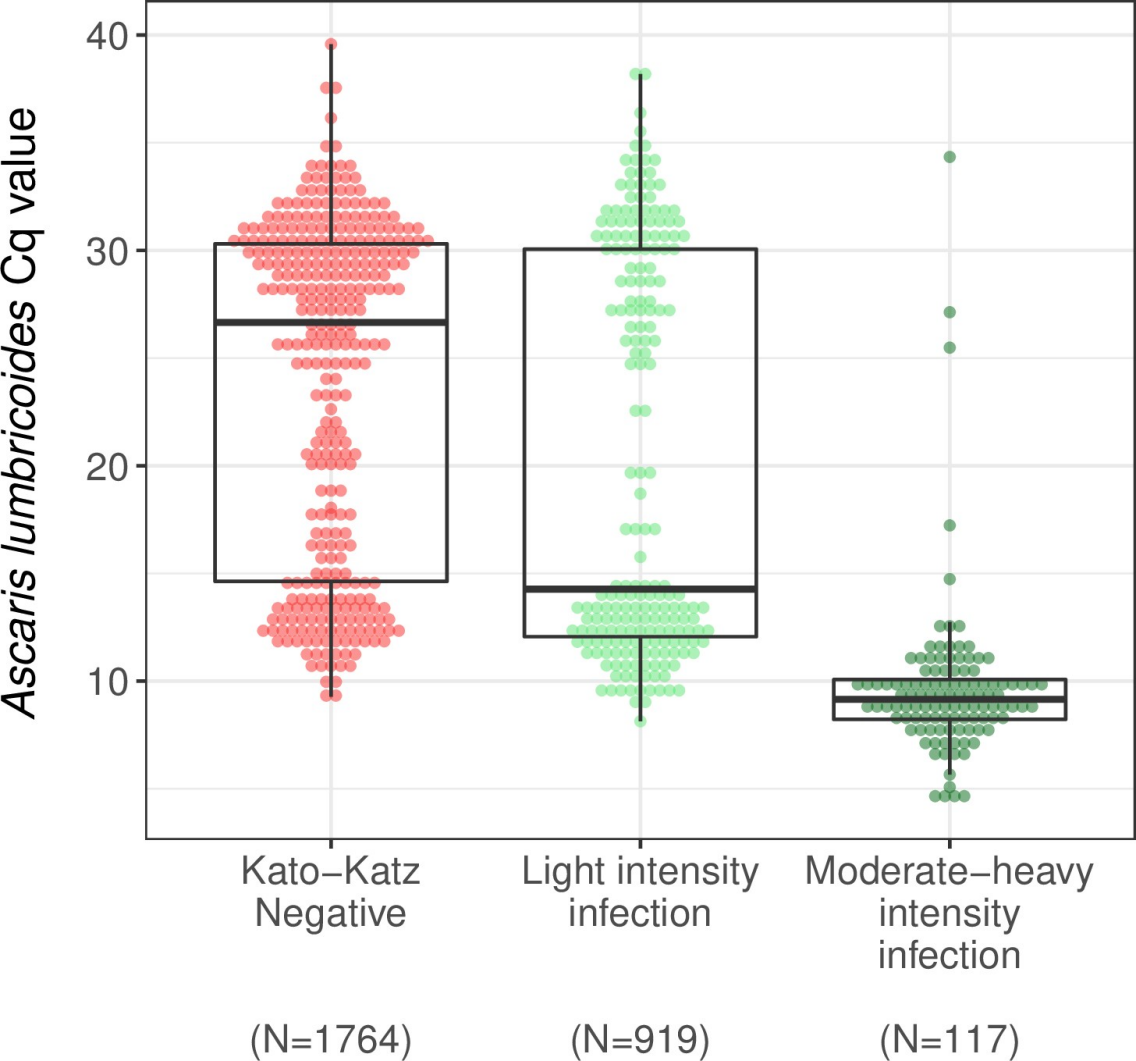
Distribution of *A*. *lumbricoides* Cq values within infection intensity categories using Double-Slide Kato-Katz. Light intensity infections were defined as < 5,000 epg for *A*. *lumbricoides*, < 1,000 epg for *T*. *trichiura*, and < 2,000 epg for hookworm; moderate intensity infections were defined as 5,000 ≤ epg < 50,000 for *A*. *lumbricoides*, 1,000 ≤ epg < 10,000 for *T*. *trichiura*, and 2,000 ≤ epg < 4,000 for hookworm; heavy intensity infections were defined as ≥ 50,000 epg for *A*. *lumbricoides*, ≥ 10,000 epg for *T*. *trichiura*, and ≥ 4,000 epg for hookworm per the World Health Organization definition.

The distribution of Cq values for *A*. *lumbricoides* was bimodal, and to a lesser extent the same pattern was present for *N*. *americanus* and *T*. *trichiura* (S10 Fig). For each STH, there was a negative, monotonic relationship between qPCR Cq values and epg estimated using double-slide Kato-Katz (Fig 3). The Kendall’s tau comparing these two quantities was -0.442 for *A*. *lumbricoides*, -0.346 for *N*. *americanus*, -0.266 for *A*. *ceylanicum*, and -0.248 for *T*. *trichiura* (for each, the p-value was < 0.001); we compared *N*. *americanus* and *A*. *ceylanicum* to any hookworm species detected by Kato-Katz. We also examined the distribution of Cq values by Kato-Katz infection intensity status for *A*. *lumbricoides* (very few children had moderate-to-heavy intensity hookworm or *T*. *trichiura* infections using Kato-Katz) (Fig 4). The distribution of Cq values was higher for children who were Kato-Katz negative and children with light intensity infections compared to those with moderate-to-heavy infections. The median Cq value was 9 (range: 4–34) among children with moderate to heavy *A*. *lumbricoides* infection, 14 (range: 8–38) among those with light intensity infection, and 27 (range: 9–40) among those who were Kato-Katz negative (t-test p-value < 0.001 for all comparisons).

In the sequencing analyses of 10 samples, we detected *Enterobius vermicularis* in one sample that was double-slide Kato-Katz negative and qPCR positive, one sample that was Kato-Katz positive and qPCR negative, and one sample that was negative using both methods (S9 Table). In addition, we identified *Giardia intestinalis* in 3 samples that were Kato-Katz positive and qPCR negative and one sample that was negative using both methods; we detected *Dientamoeba fragilis* in 3 samples that were Kato-Katz positive and qPCR negative, one sample that was Kato-Katz negative and qPCR positive, and one sample that was negative using both methods. Analysis using the QIIME2 pipeline failed to identify any reads mapping to *A*. *lumbricoides* in all seven Kato-Katz positive, qPCR negative samples. *A*. *lumbricoides* was detected in one of the two samples that were Kato-Katz negative and qPCR positive. However, this analysis failed to identify *A*. *lumbricoides* in the second sample. Because of this discrepancy, the *A*. *lumbricoides-*derived consensus sequence of the reads from the *A*. *lumbricoides-*positive sample was compared, using BLAST, to all other reads from the entire sample set. This resulted in the identification of a small number of *A*. *lumbricoides-*derived reads (n = 5, identity ≥ 97%) within the second Kato-Katz negative, qPCR positive sample, while no *A*. *lumbricoides* reads were found within the pool of reads mapping to any of the other samples. As only a single read within this Kato-Katz negative, qPCR positive sample had 100% identity with the *A*. *lumbricoides-*derived consensus sequence used as the query sequence in the BLAST analysis, QIIME2’s failure to generate an OTU from these reads follows logically, as it is likely that the 99% similarity threshold was not met for any two reads mapping to *A*. *lumbricoides* from this sample. However, the presence of *A*. *lumbricoides-*derived reads provides further support for the existence of *A*. *lumbricoides* DNA within the sample and speaks to the sensitivity of the qPCR assays used during the analysis.

## Discussion

This study compared the performance of double-slide Kato-Katz and qPCR for detecting STH infections in a large sample of 2,800 children in a setting in rural Bangladesh with biannual mass deworming administration. Consistent with prior studies in low STH infection intensity settings, using qPCR led to substantial increases in hookworm detection and moderate increases in *T*. *trichiura* detection compared to Kato-Katz [23,25,27,28,51]. Poorer performance of Kato-Katz, particularly for hookworm, is likely due to rapid disintegration of hookworm ova following stool collection [51,64]. However, unexpectedly, we found that one third of samples classified as *A*. *lumbricoides* negative by qPCR were classified as positive by Kato-Katz, and the specificity of Kato-Katz was lower for *A*. *lumbricoides* than for hookworm or *T*. *trichiura*. This discordance in classification occurred primarily among samples with epg between 10 and 100, but surprisingly, a small number of samples with *A*. *lumbricoides* epg between 1,000 and 50,000 classified as negative by qPCR (S4 Fig). Experimental testing with two qPCR assays utilizing independent targets as well as sequencing analysis of a subset of samples confirmed that samples classified as *A*. *lumbricoides* negative by qPCR contained no genomic targets for *A*. *lumbricoides*. These unexpected findings call into question the common assumption that Kato-Katz yields few false positives [22,28,57,58].

There are several potential explanations for the unexpected discrepancy between Kato-Katz and qPCR for *A*. *lumbricoides*. First, poor quality control of Kato-Katz could have caused the discrepancy. The quality control assessment found minimal differences between classification of *A*. *lumbricoides* between individual laboratory technicians and expert parasitologists (S2 Fig, S3 Fig, S8 Table), suggesting that Kato-Katz was performed with typical quality. However, the expert parasitologist that performed quality assurance also conducted Kato-Katz training; thus, if the expert mistook regularly misclassified substances in stool as *A*. *lumbricoides*, misclassification may have been passed on to technicians during training and would not have been caught during quality assurance. Second, the discrepancy could have resulted from mislabeling of stool samples. While we cannot rule out this possibility, we consider this highly unlikely; if stool samples were mislabeled, we would expect to see discrepancies for hookworm and *T*. *trichiura* as well. There was no association between the false positive rate and false negative rate (using qPCR as the gold standard) by date of Kato-Katz or date of DNA extraction, which suggests that mislabeling or other laboratory protocol violations were unlikely since both may have caused an unexpected increase in false positives/negatives at a specific point in time (S6 Fig, S7 Fig, S8 Fig, S9 Fig). Third, it is possible that improper sample storage could have resulted in STH DNA degradation that led to our discrepant findings for *A*. *lumbricoides*, however, if that was the case we would expect to also see discrepancies for other STH and in particular for hookworm, which is more fragile and is known to degrade more rapidly than *A*. *lumbricoides*. Finally, it is possible that the discrepancy was due to insufficient DNA extraction, but if this was the cause we would expect to see similarly poor performance for all qPCR assays relative to Kato-Katz results. However, qPCR had higher sensitivity than Kato-Katz for hookworm and *T*. *trichiura*. Taken together, these findings suggest that there is an alternative explanation for the *A*. *lumbricoides* discrepancy.

Misclassification of STH ova has been previously reported: *Capillaria philippinensis* or *Capillaria hepatica* ova can be mistaken for *A*. *lumbricoides* or *T*. *trichiura* ova [65,66] and *Trichostrongylus spp*. or *Schistosoma* ova can resemble hookworm ova [67,68]. Though we did identify other enteropathogens (*Giardia intestinalis* and *Dientamoeba fragilis)* when sequencing a subset of samples, in stool, these parasites do not share morphological features with most stages of *A*. *lumbricoides* [69]. We hypothesize that the substance mistaken for *A*. *lumbricoides* in Kato-Katz was most likely plant material, pollen grains, or fungal spores, all of which can resemble certain stages of *A*. *lumbricoides* [70]. Some of the sequenced stool samples contained DNA of flowering plants of the division *Magnoliophyta*, which produce pollen, and fungi of the genera *Saccharomyces* and *Candida*, which are commonly found in healthy intestinal microbiota [71] (S9 Table). We cannot definitively identify whether a different organism or substance was mistaken for *A*. *lumbricoides* because the original slides used in Kato-Katz were not preserved. Nevertheless, our findings raise concerns about the quality of the Kato-Katz procedures and underscore the advantage of using molecular methods over copromicroscopy since molecular methods do not rely on visualization of stool contents.

Overall, we found that sensitivity was lower for both double-slide Kato-Katz and qPCR than in prior studies [22,28]. A challenge in quantifying the accuracy of STH diagnostics is the lack of a gold standard measure of infection [10]. Though we used Bayesian latent class analysis, which assumes no gold standard, our estimates of sensitivity and specificity from this method should be interpreted with caution. In studies evaluating fewer than four diagnostic tests, parameters are not identifiable, and as a result parameter estimates are highly dependent upon the assumed prior distributions [52]. Our latent class models used informative priors for the specificity of each diagnostic, which we assumed was between 0.95 and 1.00 for hookworm and *T*. *trichiura*; for these STH there was agreement between results from the Bayesian latent class analysis and an analysis pooling both methods as the reference, which suggests that our assumption was appropriate. However, because of the apparent false positives for *A*. *lumbricoides* using Kato-Katz, our prior distribution for the specificity of Kato-Katz ranged from 0 to 1. Using this model with less informative priors, our estimate of sensitivity of qPCR for detecting *A*. *lumbricoides* using Bayesian latent class analysis was lower than a previous estimate for multi-parallel qPCR using the same method [28]. Using less informative priors for *A*. *lumbricoides* may have reduced the identifiability of our model and contributed to differing sensitivity and specificity estimates in this study. However, our sensitivity analysis using more informative priors for *A*. *lumbricoides* produced estimates that were similar to our primary analysis and/or closer to previously published estimates (S6 Table, S8 Table). Our model also assumed that the sensitivity and specificity of each diagnostic was constant for each study subject [72]. It is possible that sensitivity and specificity varied due to variation in STH prevalence and infection intensity, differing laboratory technicians, time from stool collection to Kato-Katz or stool archiving, or other factors; however, we expect such heterogeneity to be relatively low because our study used standardized procedures and was in a relatively small geographic area.

We observed a bimodal distribution of Cq values for *A*. *lumbricoides* and to a lesser extent for *N*. *americanus* and *T*. *trichiura*. While we cannot definitively identify the cause of this pattern, we propose two potential hypotheses. First, it is possible that the lower mode of Cq values (near Cq = 12) reflects target sequences from intact eggs, while the higher mode (near Cq = 32) reflects the presence of target sequences from samples with sub-egg quantities (i.e. sub-genome quantities) of DNA (S10 Fig). While the underlying cause was not addressed, similar distributions have been reported in prior studies [24]. Furthermore, an egg spiking study utilizing the same repeat-targeting assay employed in this study and employing the same controls and instrumentation used in this study, determined that on average, DNA extraction from a sample containing a single *A*. *lumbricoides* egg gives a Cq value of approximately 24, with the most efficient extractions commonly producing Cq values in the range of 19–22 [38]. This implies that efficiently extracted sub-egg quantities of target DNA should result in Cq values greater than 22. At nearly 250,000 copies per haploid genome, the genomic target of the employed *Ascaris* assay is more numerous than the repetitive sequences utilized as targets in any of the other assays. As such, its ability to detect trace amounts of genomic DNA is likely greater, providing an explanation for the more pronounced secondary distribution with higher Cq values. Additional research is needed to determine whether individuals with sub-egg levels of STH DNA in their stool experience clinical symptoms of infection and/or contribute to STH transmission.

A second possible explanation for the bimodal Cq distribution is that the lower mode represents fertilized eggs with diploid genomes, and the higher mode represents unfertilized eggs shed from female-only *A*. *lumbricoides* infections with haploid genomes. Unfertilized adult female *A*. *lumbricoides* worms may still shed large numbers of single-genome eggs. However, fertilized eggs undergo multiple cellular divisions during the process of embryonation, meaning that a single egg may contain many copies of the genome. Typically, under permissive environmental conditions, embryonation occurs over the course of approximately 2–3 weeks following fecal shedding, generally requiring approximately 5 days for eggs to reach the 8-cell stage [73]. When stored at colder temperatures approaching 5°C, such development is arrested [73]. Given that all stool samples collected as part of this study were placed on ice within 18 hours of sample production, it is extremely unlikely that significant embryonic development could have occurred prior to DNA extraction and qPCR testing. Therefore, while fertilized eggs would contain diploid genomes it is unlikely that significant cell division would have occurred. As such, in this study, any effect on Cq value resulting from fertilization status is likely to have been minimal, making this unlikely to account for the presence of the observed bimodal distribution.

Whether Cq values can be used to approximate intensity of STH infection is an open question. The strength of the correlation between epg and Cq values informs whether Cq value thresholds can be defined for STH infection intensity as is done using Kato-Katz [74]. Prior studies have reported moderate-to-high correlations between epg and DNA concentration for all three STH [23–25,28,75], and one study reported that Cq values correlated well with expelled adult worms following anthelminthic treatment [28]. We found moderate correlations between epg and Cq values that were lower than the correlations reported in prior studies [24,25,28]. *A*. *lumbricoides* was the only STH in this study with a sufficiently large number of samples classified as moderate-to-heavy intensity using Kato-Katz. The majority of moderate-to-heavy intensity infections had Cq values of 10 or lower (Fig 4). However, because of the misclassification of *A*. *lumbricoides*, we interpret this pattern with caution because epg counts may have been overestimated in some samples and underestimated in others. In general, a limitation of using Cq values to approximate infection intensity is that the quantity of cells per egg (and thus the copy number of a target) varies depending on fertilization status, cell number, diminution status, and the STH species [76], complicating inferences about epg using Cq values. When comparing Cq values to epg estimated from Kato-Katz, another limitation is that there is variability in the number of STH eggs secreted in stool, the number of haploid genomes present per egg, and eggs are not distributed evenly within stool or in stool samples [77,78]. Despite these limitations, Cq values represent the only quantitative output from a qPCR reaction, and as such, they may represent the best (albeit imperfect) measure for approximating infection intensity.

In settings with predominantly low STH infection intensity, highly sensitive diagnostics are needed to detect low intensity infections prior to elimination and to detect resurgent infections. Though a common critique of qPCR is its higher cost relative to Kato-Katz, comparisons of the materials required for each method suggest that costs for multi-parallel qPCR can be lower than those for Kato-Katz [25]. As pointed out by Turner et al., even if qPCR is more costly than Kato-Katz, in an elimination setting, the continued use of low sensitivity diagnostics may impede efforts to determine when STH transmission has been interrupted which may unnecessarily prolong mass deworming administration. The cost of prolonged MDA is likely to exceed that of a more costly diagnostic [79]. Our results support the use of qPCR in elimination settings due to its higher sensitivity and specificity. In addition, importantly, using qPCR instead of Kato-Katz in intervention trials can also reduce misclassification and bias of intervention effect estimates [80].

## Conclusion

Kato-Katz remains a highly cost-effective, feasible diagnostic method appropriate for certain uses cases, such as identifying where to perform MDA, determining the frequency of MDA, and assessing progress towards program goals [30]. Prior to this study, the greatest disadvantage of Kato-Katz was typically considered to be its low sensitivity; here we show that not only was sensitivity lower for Kato-Katz than for qPCR, but also in our setting, Kato-Katz had low specificity for *A*. *lumbricoides*. Though we were not able to definitively determine the cause of the apparent *A*. *lumbricoides* misclassification using Kato-Katz, regardless of the cause, our results highlight an inherent limitation of Kato-Katz. In addition, our results echo conclusions of other recent studies that qPCR may be more appropriate for use cases such as identifying whether STH transmission has been interrupted and confirming sustained transmission interruption due to its higher sensitivity in low STH intensity settings [30].

## Supporting information

S1 Checklist STARD Checklist. (PDF)

S2 Checklist STARD Bayesian Latent Class Models Analysis Checklist. (PDF)

S1 Table Percent agreement between the original Kato-Katz technician and the senior parasitologist by days elapsed between each assessment. (PDF)

S2 Table Target information, sequences, manufacturers, and relevant references for all qPCR primers and probes used during this study. (XLSX)

S3 Table Results of plasmid control testing for each assay. For a given plate, in the event that any concentration of control plasmid was 3 or more standard deviations greater than the mean (calculated from all experimental plates), the plate was rerun in its entirety and the results of initial testing were considered invalid.
(XLSX)

S4 Table Primer sequences used for 18S SSU sequencing. (PDF)

S5 Table Prior distributions used in Bayesian latent class analysis models. (PDF)

S6 Table Alternative prior distributions used in sensitivity analysis for Bayesian latent class analysis models for *A*. *lumbricoides*. (PDF)

S7 Table Kappa statistics comparing the original classification of a single slide as having any STH ova between individual Kato-Katz technicians and expert parasitologists. (PDF)

S8 Table Sensitivity analysis using alternative prior distributions in Bayesian latent class analysis models for *A*. *lumbricoides*. (PDF)

S9 Table Taxonomic assignments for OTUs resulting from sequencing analysis with the Qiime2 pipeline. Following de-multiplexing by barcode, OTUs underwent taxonomic assignment. For each sample, the number of interlaced read pairs mapping to each taxonomic assignment is provided.
(XLSX)

S1 Text Bayesian latent class model specification. (PDF)

S1 Fig Participant flow diagram. (PDF)

S2 Fig Percent agreement between laboratory technicians and expert technicians in STH positive/negative status in individual slides. (PDF)

S3 Fig Mean difference in Kato-Katz single slide egg count between laboratory technician and expert counter among samples that were positive by either the laboratory technician or expert. (PDF)

S4 Fig Distribution of *A*. *lumbricoides* eggs per gram classified by concordance status between Kato-Katz and qPCR. (PDF)

S5 Fig Probability that a stool sample was classified as positive for *A*. *lumbricoides* using Kato-Katz among those classified as negative by qPCR by individual Kato-Katz technician. (PDF)

S6 Fig Cumulative probability that a stool sample was classified as positive for *A*. *lumbricoides* using Kato-Katz among those classified as negative by qPCR by date of Kato-Katz. (PDF)

S7 Fig Cumulative probability that a stool sample was classified as positive for *A*. *lumbricoides* using Kato-Katz among those classified as negative by qPCR by date of DNA extraction. (PDF)

S8 Fig Cumulative probability that a stool sample was classified as negative using Kato-Katz among those classified as positive by qPCR by date of Kato-Katz. (PDF)

S9 Fig Cumulative probability that a stool sample was classified as negative using Kato-Katz among those classified as positive by qPCR by date of DNA extraction. (PDF)

S10 Fig Distribution of Cq values for each soil-transmitted helminth. (PDF)
