# Supplementary material for: Comparison of multi-parallel qPCR and double-slide Kato-Katz for detection of soil-transmitted helminth infection among children in rural Bangladesh
Source: PLoS Negl Trop Dis. 2020 Apr 24;14(4):e0008087. doi: 10.1371/journal.pntd.0008087 (PMC7202662; doi:10.1371/journal.pntd.0008087)
Supplement: S2 Checklist — (PDF) [file pntd.0008087.s002.pdf]

Comparison of multi-parallel qPCR and double-slide Kato-Katz for detection of soil-transmitted helminth infection among children in rural Bangladesh

S2 Checklist: STARD Bayes Latent Class Models Checklist

Section & Topic	No	Item	Reported on page #
**TITLE OR ABSTRACT**			
	**1**	Identification as a study of diagnostic accuracy, using at least one measure of accuracy (such as sensitivity, specificity, predictive values, or AUC) **and Bayesian latent class models**	Abstract
**ABSTRACT**			
	**2**	Structured summary of study design, methods, results, and conclusions (for specific guidance, see STARD for Abstracts)	Abstract
**INTRODUCTION**			
	**3**	Scientific and clinical background, including the intended use and clinical role of the **tests under evaluation**	Introduction, paragraph 2-4
	**4**	Study objectives and hypotheses, **such as estimation of diagnostic accuracy of the tests for a defined purpose through BLCM**	Introduction, paragraph 5
**METHODS**			
Study design	**5**	Whether data collection was planned before the **tests** were performed (prospective study) or after (retrospective study)	Methods, paragraph 1-4
Participants	**6**	Eligibility criteria **and description of the source population**	Methods, paragraph 1
	**7**	On what basis potentially eligible participants were identified (such as symptoms, results from previous tests, inclusion in registry)	Methods, paragraph 1
	**8**	Where and when potentially eligible participants were identified (setting, location and dates)	Methods, paragraph 1
	**9**	Whether participants formed a consecutive, random or convenience series	Methods, paragraph 1
Test methods	**10**	**Description of the tests under evaluation**, in sufficient detail to allow replication, **and/or cite references**	Methods, paragraph 3-7
	**11**	Rationale for choosing the **tests under evaluation in relation to their purpose**	Introduction, paragraph 2-4
	**12**	Definition of and rationale for test positivity cut-offs or result categories of **the tests under evaluation**, distinguishing pre-specified from exploratory	Methods, paragraph 3
	**13**	Whether clinical information was available to the performers or readers of **the tests under evaluation**	Methods, paragraph 3-4
Analysis	**14a**	**BLCM model** for estimating measures of diagnostic accuracy	Methods, paragraph 14
	**14b**	**Definition and rationale of prior information and sensitivity analysis**	Methods, paragraph 14
	**15**	How indeterminate results **of the tests under evaluation** were handled	Methods, paragraph 4, 6
	**16**	How missing data **of the tests under evaluation** were handled	N/A
	**17**	Any analyses of variability in diagnostic accuracy, distinguishing pre-specified from exploratory	Methods, paragraph 12-14
	**18**	Intended sample size and how it was determined	Methods, paragraph 1
**RESULTS**			
Participants	**19**	Flow of participants, using a diagram	S3 Appendix
	**20**	Baseline demographic and clinical characteristics of participants	Methods, paragraph 7; Results, paragraph 1
	**21**	**Not applicable: the distribution of the targeted conditions is unknown, hence the use of BLCM**	
	**22**	Time interval and any clinical interventions between **the tests under evaluation**	Methods, paragraph 2-3
Test results	**23**	Cross tabulation of the **tests' results (or for continuous tests results their distribution by infection stage)**	Results, paragraph 3; Table 3

	**24**	Estimates of diagnostic accuracy **under alternative prior specification** and their precision (such as 95% **credible/probability intervals**)	S3 Appendix
	**25**	Any adverse events from performing **the tests under evaluation**	N/A
**DISCUSSION**			
	**26**	Study limitations, including sources of potential bias, statistical uncertainty, and generalisability	Discussion, paragraph 2, 4, 7
	**27**	Implications for practice, including the intended use and clinical role of **the tests under evaluation in relevant settings (clinical, research, surveillance etc.)**	Discussion, paragraph 1, 8; Conclusion
**OTHER INFORMATION**			
	**28**	Registration number and name of registry	Methods, paragraph 1
	**29**	Where the full study protocol can be accessed	Methods, paragraph 1
	**30**	Sources of funding and other support; role of funders	Acknowledgements

STARD - BLCM

STARD-BLCM stands for “Standards for the Reporting of Diagnostic accuracy studies that use Bayesian Latent Class Models” and is a modification of the STARD statement (which was recently updated to STARD2015). STARD-BLCM aims to facilitate improved quality of reporting for diagnostic accuracy studies that use Bayesian latent class models in the absence of a reference standard. The proposed modifications are relevant to both Bayesian and frequentist estimation methods but the focus is on the former.

More information for STARD (STARD2015) can be found at: http://www.equator-network.org/reporting-guidelines/stard

More information for STARD-BLCM can be found at: http://www.equator-network.org/reporting-guidelines/stard-blcm
