# Supplementary material for: Comparison of multi-parallel qPCR and double-slide Kato-Katz for detection of soil-transmitted helminth infection among children in rural Bangladesh
Source: PLoS Negl Trop Dis. 2020 Apr 24;14(4):e0008087. doi: 10.1371/journal.pntd.0008087 (PMC7202662; doi:10.1371/journal.pntd.0008087)
Supplement: S1 Table — (PDF) [file pntd.0008087.s003.pdf]

Comparison of multi-parallel qPCR and double-slide Kato-Katz for detection of soil-transmitted helminth infection among children in rural Bangladesh

S1 Table. Percent agreement between the original Kato-Katz technician and the senior parasitologist by days elapsed between each assessment

Days	Ascaris	Hookworm	Trichuris
0	1.00	1.00	1.00
1	0.91	0.33	0.81
2-4	0.94	0.11	0.91
>4	0.88	0.00	0.83
Overall	0.92	0.20	0.86
