# Supplementary material for: Comparison of multi-parallel qPCR and double-slide Kato-Katz for detection of soil-transmitted helminth infection among children in rural Bangladesh
Source: PLoS Negl Trop Dis. 2020 Apr 24;14(4):e0008087. doi: 10.1371/journal.pntd.0008087 (PMC7202662; doi:10.1371/journal.pntd.0008087)
Supplement: S4 Table — (PDF) [file pntd.0008087.s006.pdf]

Comparison of multi-parallel qPCR and double-slide Kato-Katz for detection of soil-transmitted helminth infection among children in rural Bangladesh

S4 Table. Primer sequences used for 18S SSU sequencing

Primer Name	Primer Sequence (5’ → 3’) [a]
Read 1 Sequencing Primer	TATCGCCGTTCGGTACACACCGCCCGTC
Read 2 Sequencing Primer	AGTCAGCCAGCATGATCCTTCTGCAGGTTCACCTAC
Index Sequence Primer	GTAGGTGAACCTGCAGAAGGATCATGCTGACTGACT
Forward Primer for PCR	AATGATACGGCGACCACCGAGATCTACACTATCGCCGTTCGGTACACACCGCCCGTC
Reverse Primer for PCR #1	CAAGCAGAAGACGGCATACGAGATACGAGACTGATTAGTCAGTCAGCATGATCCTTCTGCAGGTTCACCTAC
Reverse Primer for PCR #2	CAAGCAGAAGACGGCATACGAGATGCTGTACGGATTAGTCAGTCAGCATGATCCTTCTGCAGGTTCACCTAC
Reverse Primer for PCR #3	CAAGCAGAAGACGGCATACGAGATATCACCAGGTGTAGTCAGTCAGCATGATCCTTCTGCAGGTTCACCTAC
Reverse Primer for PCR #4	CAAGCAGAAGACGGCATACGAGATTGGTCAACGATAAGTCAGTCAGCATGATCCTTCTGCAGGTTCACCTAC
Reverse Primer for PCR #5	CAAGCAGAAGACGGCATACGAGATATCGCACAGTAAAGTCAGTCAGCATGATCCTTCTGCAGGTTCACCTAC
Reverse Primer for PCR #6	CAAGCAGAAGACGGCATACGAGATGTCGTGTAGCCTAGTCAGTCAGCATGATCCTTCTGCAGGTTCACCTAC
Reverse Primer for PCR #7	CAAGCAGAAGACGGCATACGAGATAGCGGAGGTTAGAGTCAGTCAGCATGATCCTTCTGCAGGTTCACCTAC
Reverse Primer for PCR #8	CAAGCAGAAGACGGCATACGAGATTACAGCGCATACAGTCAGTCAGCATGATCCTTCTGCAGGTTCACCTAC
Reverse Primer for PCR #9	CAAGCAGAAGACGGCATACGAGATAATTGTGTCGGAAGTCAGTCAGCATGATCCTTCTGCAGGTTCACCTAC
Reverse Primer for PCR #10	CAAGCAGAAGACGGCATACGAGATTGCATACACTGGAGTCAGTCAGCATGATCCTTCTGCAGGTTCACCTAC
Mammal Blocking Primer	GCCCGTCGCTACTACCGATTGG/ideoxyI//ideoxyI//ideoxyI//ideoxyI//ideoxyI/TTAGTGAGGCCCT/3SpC3/

[a] The barcode sequence in each reverse primer is underlined.
