# Supplementary material for: Comparison of multi-parallel qPCR and double-slide Kato-Katz for detection of soil-transmitted helminth infection among children in rural Bangladesh
Source: PLoS Negl Trop Dis. 2020 Apr 24;14(4):e0008087. doi: 10.1371/journal.pntd.0008087 (PMC7202662; doi:10.1371/journal.pntd.0008087)
Supplement: S5 Table — (PDF) [file pntd.0008087.s007.pdf]

Comparison of multi-parallel qPCR and double-slide Kato-Katz for detection of soil-transmitted helminth infection among children in rural Bangladesh

S5 Table. Prior distributions used in Bayesian latent class analysis models

	A. lumbricoides	**Hookworm**	***T. trichiura***
Prevalence	Beta distribution with shape parameters α=1, β=1	Beta distribution with shape parameters α=1, β=1	Beta distribution with shape parameters α=1, β=1
Kato-Katz sensitivity	Beta distribution with shape parameters α=1, β=1	Beta distribution with shape parameters α=3, β=3	Beta distribution with shape parameters α=3, β=3
Kato-Katz specificity	Beta distribution with shape parameters α=1, β=1	Uniform distribution with minimum=0.95, maximum=1	Uniform distribution with minimum=0.95, maximum=1
qPCR sensitivity	Uniform distribution with minimum=0.60, maximum=1	Uniform distribution with minimum=0.80, maximum=1	Uniform distribution with minimum=0.80, maximum=1
qPCR specificity	Uniform distribution with minimum=0.95, maximum=1	Uniform distribution with minimum=0.95, maximum=1	Uniform distribution with minimum=0.95, maximum=1

Due to the discrepancy between Kato-Katz and qPCR results for *A. lumbricoides*, we chose non-informative prior distributions for Kato-Katz sensitivity and specificity and a less informative prior distribution for qPCR sensitivity.
