# Supplementary material for: Comparison of multi-parallel qPCR and double-slide Kato-Katz for detection of soil-transmitted helminth infection among children in rural Bangladesh
Source: PLoS Negl Trop Dis. 2020 Apr 24;14(4):e0008087. doi: 10.1371/journal.pntd.0008087 (PMC7202662; doi:10.1371/journal.pntd.0008087)
Supplement: S6 Table — (PDF) [file pntd.0008087.s008.pdf]

Comparison of multi-parallel qPCR and double-slide Kato-Katz for detection of soil-transmitted helminth infection among children in rural Bangladesh

S6 Table. Alternative prior distributions used in sensitivity analysis for Bayesian latent class analysis models for *A. lumbricoides*

	**Sensitivity analysis 1**:<br><br>More informative prior for Kato-Katz sensitivity and specificity	**Sensitivity analysis 2:**<br><br>More informative prior for qPCR sensitivity	**Sensitivity analysis 3:**<br><br>More informative prior for sensitivity and specificity of both Kato-Katz and qPCR
Prevalence	Beta distribution with shape parameters α=1, β=1	Beta distribution with shape parameters α=1, β=1	Beta distribution with shape parameters α=1, β=1
Kato-Katz sensitivity	Beta distribution with shape parameters α=3, β=3	Beta distribution with shape parameters α=3, β=3	Beta distribution with shape parameters α=3, β=3
Kato-Katz specificity	Uniform distribution with minimum=.5, maximum=1	Beta distribution with shape parameters α=1, β=1	Uniform distribution with minimum=.5, maximum=1
qPCR sensitivity	Uniform distribution with minimum=0.6, maximum=1	Uniform distribution with minimum=0.8, maximum=1	Uniform distribution with minimum=0.8, maximum=1
qPCR specificity	Uniform distribution with minimum=0.95, maximum=1	Uniform distribution with minimum=0.95, maximum=1	Uniform distribution with minimum=0.95, maximum=1
