# Supplementary material for: Comparison of multi-parallel qPCR and double-slide Kato-Katz for detection of soil-transmitted helminth infection among children in rural Bangladesh
Source: PLoS Negl Trop Dis. 2020 Apr 24;14(4):e0008087. doi: 10.1371/journal.pntd.0008087 (PMC7202662; doi:10.1371/journal.pntd.0008087)
Supplement: S7 Table — (PDF) [file pntd.0008087.s009.pdf]

Comparison of multi-parallel qPCR and double-slide Kato-Katz for detection of soil-transmitted helminth infection among children in rural Bangladesh

S7 Table. Kappa statistics comparing the original classification of a single slide as having any STH ova between individual Kato-Katz technicians and expert parasitologists

	Ascaris		**Hookworm**		***Trichuris***	
Technician	Kappa statistic	p-value	Kappa statistic	p-value	Kappa statistic	p-value
1	0.83	<0.001	--	--	--	--
2	0.90	<0.001	0.18	0.004	0.79	<0.001
3	1.00	<0.001	--	--	0.66	<0.001
4	0.91	<0.001	0	--	1.00	<0.001

Some values were not estimated (as indicated by --) because neither the technician nor the expert parasitologist identified any ova. The agreement is likely lower for hookworm because experienced parasitologists examined slides up to a few days after laboratory technicians, and hookworm ova may have begun to disintegrate by that time. In the primary study, in the subset of samples for which quality control was performed on the same day, the agreement for hookworm was high (Ercumen et al., 2019. https://doi.org/10.1371/journal.pntd.0007323).
