# Supplementary material for: Comparison of multi-parallel qPCR and double-slide Kato-Katz for detection of soil-transmitted helminth infection among children in rural Bangladesh
Source: PLoS Negl Trop Dis. 2020 Apr 24;14(4):e0008087. doi: 10.1371/journal.pntd.0008087 (PMC7202662; doi:10.1371/journal.pntd.0008087)
Supplement: S8 Table — (PDF) [file pntd.0008087.s010.pdf]

Comparison of multi-parallel qPCR and double-slide Kato-Katz for detection of soil-transmitted helminth infection among children in rural Bangladesh

S8 Table. Sensitivity analysis using alternative prior distributions in Bayesian latent class analysis models for *A. lumbricoides*

		Kato-Katz		qPCR	
**Analysis**	**Description of priors**	**Sensitivity (95% BCI)**	**Specificity (95% BCI)**	**Sensitivity (95% BCI)**	**Specificity (95% BCI)**
Primary analysis result	Noninformative priors	49 (34, 64)	68 (61, 77)	79 (61, 99)	97 (95, 100)
Sensitivity analysis 1	More informative prior for Kato-Katz sensitivity and specificity	49 (34, 64)	68 (62, 76)	80 (61, 99)	97 (95, 100)
Sensitivity analysis 2	More informative prior for qPCR sensitivity	49 (38, 59)	67 (63, 71)	90 (80, 99)	97 (95, 100)
Sensitivity analysis 3	More informative prior for sensitivity and specificity of both Kato-Katz and qPCR	49 (39, 59)	67 (63, 71)	90 (80, 99)	97 (95, 100)
