# Supplementary material for: Comparison of multi-parallel qPCR and double-slide Kato-Katz for detection of soil-transmitted helminth infection among children in rural Bangladesh
Source: PLoS Negl Trop Dis. 2020 Apr 24;14(4):e0008087. doi: 10.1371/journal.pntd.0008087 (PMC7202662; doi:10.1371/journal.pntd.0008087)
Supplement: S2 Fig — (PDF) [file pntd.0008087.s014.pdf]

Comparison of multi-parallel qPCR and double-slide Kato-Katz for detection of soil-transmitted helminth infection among children in rural Bangladesh

S2 Figure. Percent agreement between laboratory technicians and expert technicians in STH positive/negative status in individual slides

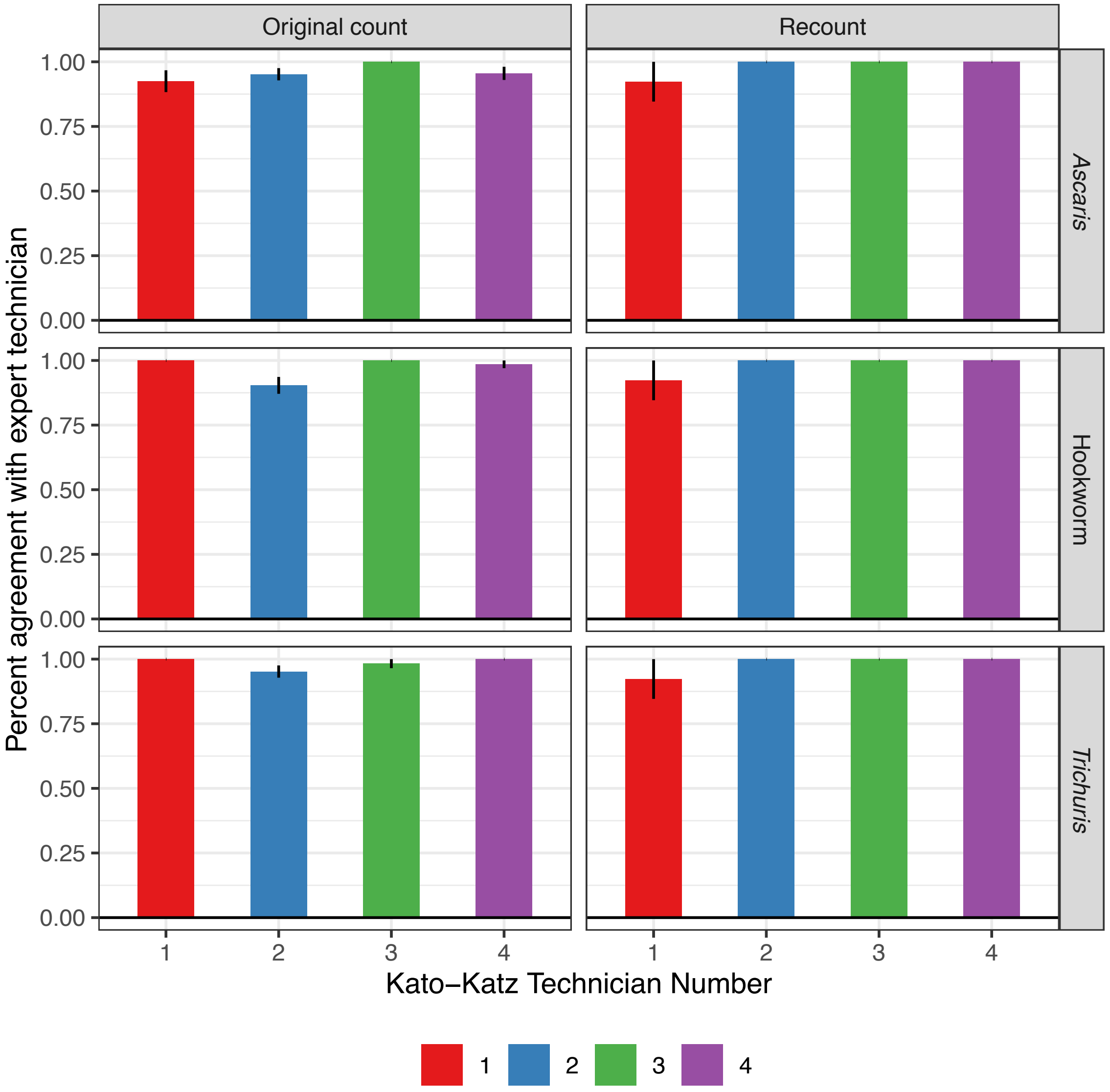

Each bar indicates the percent agreement in STH positive/negative status between an individual technician and an expert technician on a single slide reading. The black vertical line indicates the 95% confidence interval.
