# Supplementary material for: Comparison of multi-parallel qPCR and double-slide Kato-Katz for detection of soil-transmitted helminth infection among children in rural Bangladesh
Source: PLoS Negl Trop Dis. 2020 Apr 24;14(4):e0008087. doi: 10.1371/journal.pntd.0008087 (PMC7202662; doi:10.1371/journal.pntd.0008087)
Supplement: S3 Fig — (PDF) [file pntd.0008087.s015.pdf]

Comparison of multi-parallel qPCR and double-slide Kato-Katz for detection of soil-transmitted helminth infection among children in rural Bangladesh

S3 Figure. Mean difference in Kato-Katz single slide egg count between laboratory technician and expert counter among samples that were positive by either the laboratory technician or expert

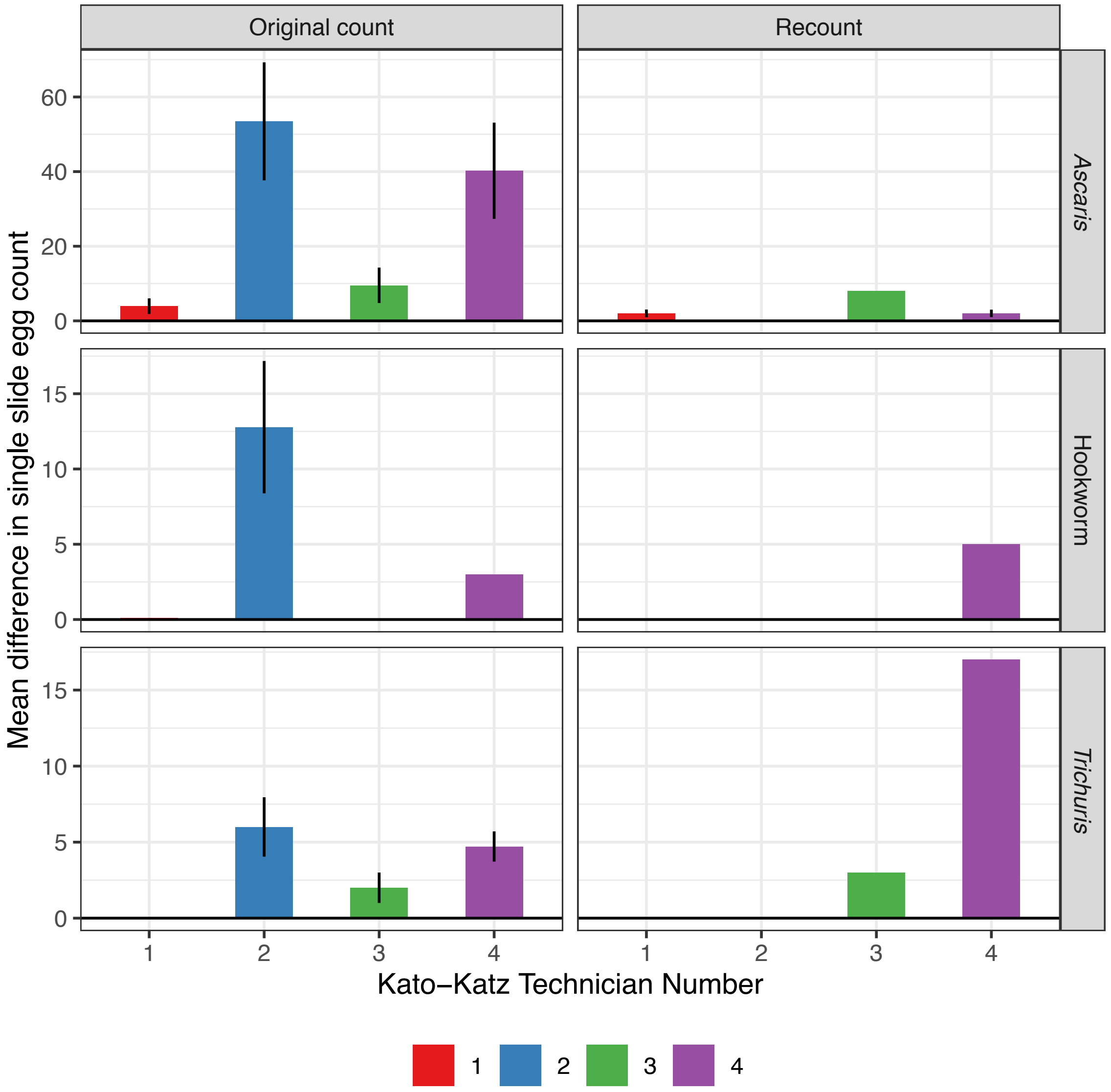

Each bar indicates the mean difference in single slide egg counts between an individual technician and an expert technician. The black vertical line indicates the 95% confidence interval.
