# Supplementary material for: Comparison of multi-parallel qPCR and double-slide Kato-Katz for detection of soil-transmitted helminth infection among children in rural Bangladesh
Source: PLoS Negl Trop Dis. 2020 Apr 24;14(4):e0008087. doi: 10.1371/journal.pntd.0008087 (PMC7202662; doi:10.1371/journal.pntd.0008087)

***Comparison of multi-parallel qPCR and double-slide Kato-Katz for detection of soil-transmitted helminth infection among children in rural Bangladesh***

**S4 Figure. Distribution of *A. lumbricoides* eggs per gram classified by concordance status between Kato-Katz and qPCR**

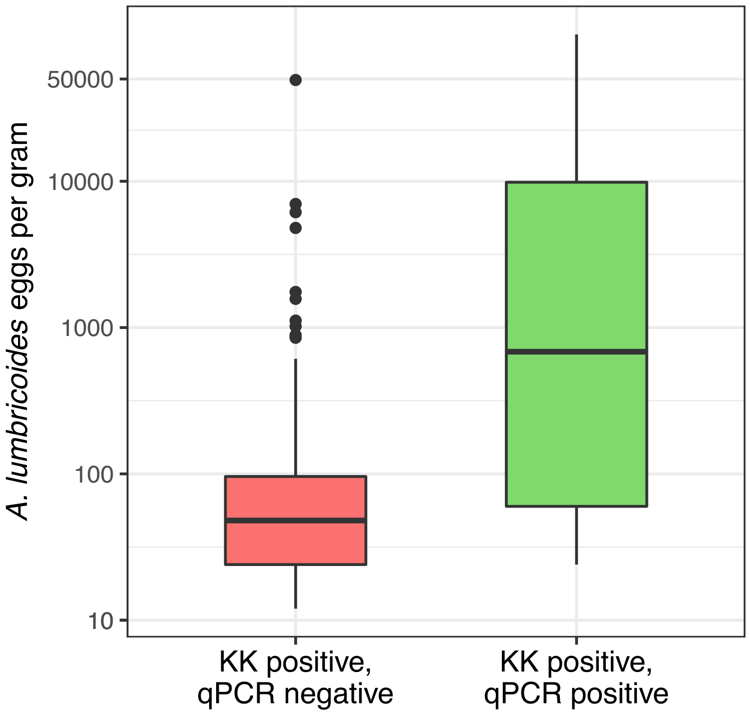

Supplement: S4 Fig — (PDF) [file pntd.0008087.s016.pdf]
