# Supplementary material for: Comparison of multi-parallel qPCR and double-slide Kato-Katz for detection of soil-transmitted helminth infection among children in rural Bangladesh
Source: PLoS Negl Trop Dis. 2020 Apr 24;14(4):e0008087. doi: 10.1371/journal.pntd.0008087 (PMC7202662; doi:10.1371/journal.pntd.0008087)
Supplement: S6 Fig — (PDF) [file pntd.0008087.s018.pdf]

Comparison of multi-parallel qPCR and double-slide Kato-Katz for detection of soil-transmitted helminth infection among children in rural Bangladesh

S6 Figure. Cumulative probability that a stool sample was classified as positive for *A. lumbricoides* using Kato-Katz among those classified as negative by qPCR by date of Kato-Katz

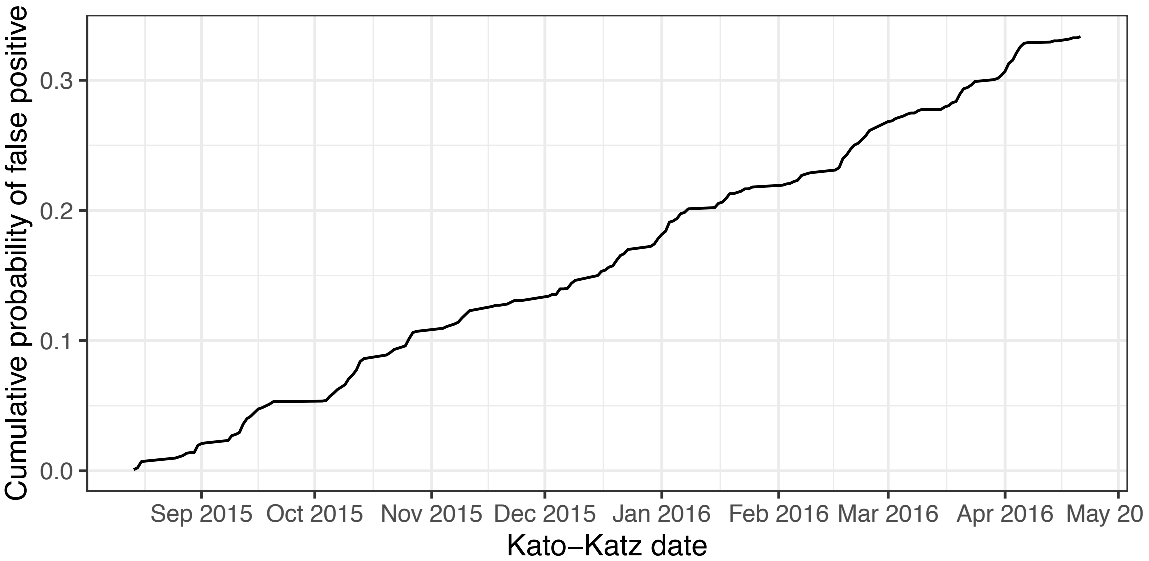

Data were not included for hookworm and *Trichuris* since only 1% of samples were classified as positive by Kato-Katz among those classified as negative by qPCR for those STH.
