# Supplementary material for: Comparison of multi-parallel qPCR and double-slide Kato-Katz for detection of soil-transmitted helminth infection among children in rural Bangladesh
Source: PLoS Negl Trop Dis. 2020 Apr 24;14(4):e0008087. doi: 10.1371/journal.pntd.0008087 (PMC7202662; doi:10.1371/journal.pntd.0008087)

Comparison of multi-parallel qPCR and double-slide Kato-Katz for detection of soil-transmitted helminth infection among children in rural Bangladesh

S8 Figure. Cumulative probability that a stool sample was classified as negative using Kato-Katz among those classified as positive by qPCR by date of Kato-Katz

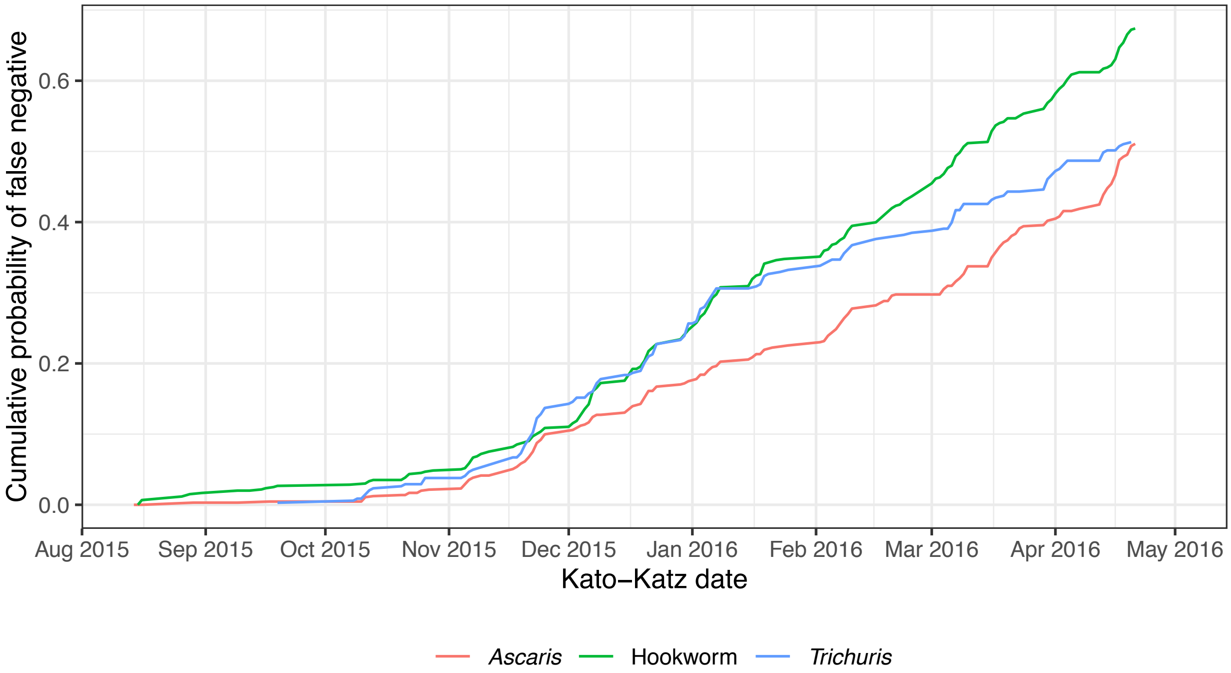

Supplement: S8 Fig — (PDF) [file pntd.0008087.s020.pdf]
