# Supplementary material for: Comparison of multi-parallel qPCR and double-slide Kato-Katz for detection of soil-transmitted helminth infection among children in rural Bangladesh
Source: PLoS Negl Trop Dis. 2020 Apr 24;14(4):e0008087. doi: 10.1371/journal.pntd.0008087 (PMC7202662; doi:10.1371/journal.pntd.0008087)
Supplement: S9 Fig — (PDF) [file pntd.0008087.s021.pdf]

Comparison of multi-parallel qPCR and double-slide Kato-Katz for detection of soil-transmitted helminth infection among children in rural Bangladesh

S9 Figure. Cumulative probability that a stool sample was classified as negative using Kato-Katz among those classified as positive by qPCR by date of DNA extraction

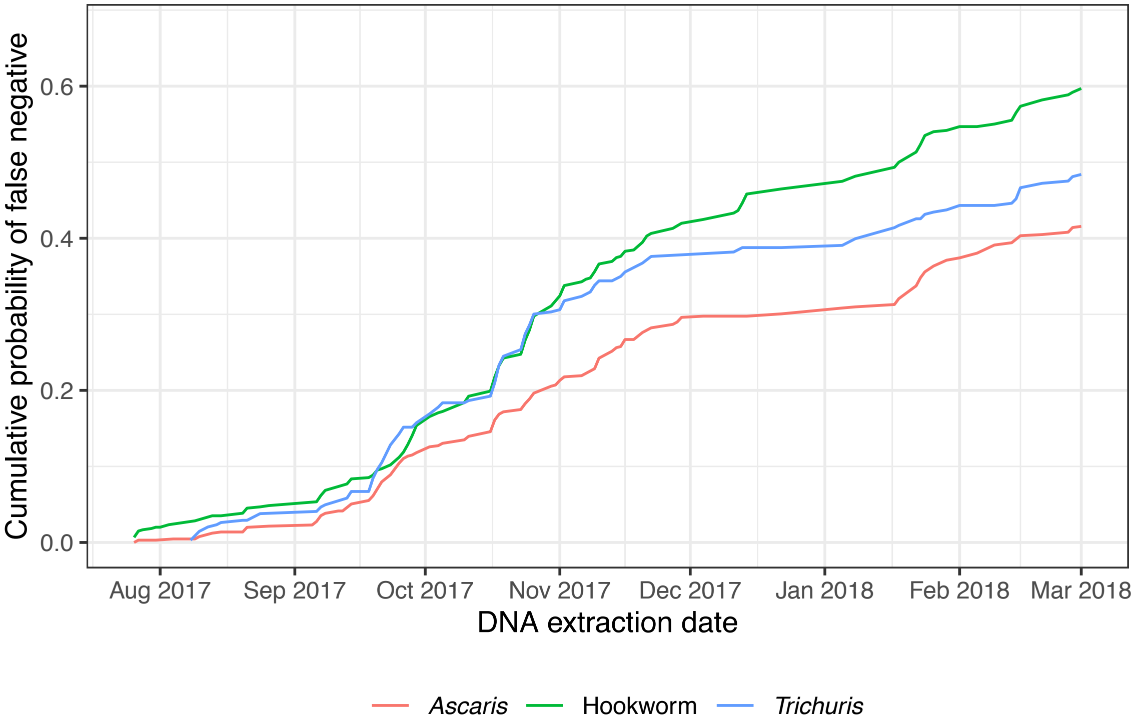

The date of DNA extraction was not recorded for the last 242 samples; we imputed the date for those samples as 1 day later than the last recorded date.
